# Zika Virus-Induced Metabolic Reprogramming Drives Lipid Droplet Biogenesis, Promoting Viral Replication and Ocular Pathogenesis

**DOI:** 10.3390/cells15090817

**Published:** 2026-04-30

**Authors:** Prince Kumar, Jieon Kim, Nikhil Deshmukh, Pawan Kumar Singh

**Affiliations:** Department of Ophthalmology, Mason Eye Institute, University of Missouri School of Medicine, 1 Hospital Dr, Columbia, MO 65212, USA

**Keywords:** Zika virus, trabecular meshwork, AMPK, lipid metabolism, energy metabolism, fatty acids, lipid droplet, β-oxidation, glaucoma

## Abstract

**Highlights:**

**What are the main findings?**

- Zika virus (ZIKV) reprograms trabecular meshwork cell metabolism by activating AMPK signaling and promoting lipid droplet (LD) biogenesis.
- Fatty acid (FA) metabolism regulates infection, where saturated FAs enhance and unsaturated FAs suppress ZIKV replication by affecting viral entry.

**What are the implications of the main findings?**

- Host metabolic pathways (AMPK, LD, and FA metabolism) are key regulators of ZIKV ocular infection and pathogenesis.
- These pathways represent potential therapeutic targets for preventing or treating ZIKV-associated ocular complications.

**Abstract:**

Zika virus (ZIKV) remains a significant global public health threat due to its association with severe neurological and ocular abnormalities, including microcephaly and congenital glaucoma in infants. Viruses often exploit host metabolic programs, such as energy and lipid metabolism, to support their replication. However, how ZIKV-driven metabolic reprogramming affects the anterior segment of the eye, especially trabecular meshwork (TM) cells, remains poorly defined. In this study, we investigated the roles of AMP-activated protein kinase (AMPK) signaling, fatty acid (FA) metabolism, and lipid droplet (LD) biogenesis in ZIKV-induced ocular pathogenesis using primary human TM cells and an IFNAR1-deficient mouse model. ZIKV infection triggered time-dependent activation of the LKB1-AMPK-ACC signaling axis and significantly increased LD accumulation. Pharmacological activation of AMPK suppressed viral replication, whereas its inhibition enhanced infection, highlighting an antiviral role for AMPK signaling. In contrast, ZIKV promoted LD biogenesis, and inhibition of DGAT1 reduced both LD formation and viral replication, indicating a proviral role for LDs. Modulation of FA metabolism further revealed differential effects on ZIKV infection: saturated FA (palmitate) enhanced viral replication, whereas inhibition of FA oxidation with etomoxir reduced infection. Conversely, unsaturated FAs (oleate and linoleate) suppressed viral replication, in part by impairing viral binding and entry. Collectively, these findings show that ZIKV reshapes host metabolic pathways in TM by differentially engaging AMPK signaling, FA metabolism, and LD biogenesis to promote viral replication and spread in ocular tissue. Targeting these metabolic pathways may offer promising therapeutic avenues for preventing and/or treating ZIKV-associated ocular complications.

## 1. Introduction

Zika virus (ZIKV), a mosquito-borne flavivirus, remains a global health concern because of its link to serious neurological, auditory, and ocular complications [1,2,3]. Although infection in adults is typically mild or asymptomatic, congenital exposure can lead to profound developmental abnormalities, including severe ocular defects, with or without microcephaly [2,4,5]. ZIKV demonstrates marked tropism for key ocular tissues in both the anterior and posterior segments of the eye [4,5,6]. Reported congenital ocular manifestations include retinal damage, optic nerve defects, vascular abnormalities, and congenital glaucoma [5,6,7,8,9,10]. Despite growing clinical and experimental evidence linking ZIKV to eye diseases, the underlying molecular mechanisms driving ZIKV-induced ocular pathology remain unclear, particularly in trabecular meshwork (TM) cells of the anterior segment. Given the essential role of the TM in regulating aqueous humor outflow and intraocular pressure (IOP), its dysfunction may represent a key mechanism contributing to glaucomatous pathology in ZIKV-infected infants.

Viruses are obligate intracellular parasites that rely heavily on host metabolic machinery to complete their life cycle [11]. Increasing evidence indicates that host metabolism actively regulates viral replication and antiviral responses, rather than serving merely as a passive substrate [12]. Among these metabolic regulators, energy and lipid metabolism have emerged as critical interfaces between host–virus interactions. AMP-activated protein kinase (AMPK) is a highly conserved energy sensor that coordinates cellular responses to metabolic stress by promoting catabolic processes, such as fatty acid β-oxidation, while inhibiting anabolic pathways, including lipogenesis, primarily through downstream targets such as acetyl-CoA carboxylase (ACC) [13,14]. In addition to metabolic regulation, AMPK also modulates innate immune signaling, positioning it at the intersection of metabolism and antiviral defense [15,16]. Consistent with this, AMPK activation has been shown to limit the replication of several RNA viruses, whereas its inhibition may favor viral replication [16,17]. Notably, our observations suggest that ZIKV differentially modulates AMPK signaling in ocular cells: it suppresses the AMPK-ACC axis in retinal vascular endothelial cells [18] while activating it in retinal pigmented epithelium (unpublished data), suggesting cell-type-specific metabolic reprogramming during infection. However, how ZIKV modulates AMPK activity in TM cells of the anterior segment remains unknown.

Lipid metabolism plays a central role in shaping viral infection dynamics. Fatty acids serve not only as energy sources but also as essential building blocks for cellular membranes and signaling molecules, making them indispensable for viral replication and assembly [19,20,21]. Many viruses reprogram host fatty acid synthesis and β-oxidation to ensure a steady supply of these resources, while host cells attempt to counterbalance this metabolic shift as part of the antiviral response. In parallel, lipid droplets (LDs) have emerged as dynamic organelles with active roles during infection. Once viewed as simple fat storage depots, LDs are now recognized as metabolically active hubs involved in lipid trafficking, protein sequestration, and immune regulation [22,23]. Many RNA viruses, including flaviviruses, induce LD biogenesis and exploit them as platforms for replication, lipid reservoirs for membrane remodeling, and source of energy through β-oxidation [21,24,25]. AMPK lies at the center of this metabolic network, regulating fatty acid metabolism and LD dynamics, thereby influencing both viral replication and host defense. Although AMPK activation is generally associated with suppression of lipid synthesis and reduced viral replication, several viruses have evolved strategies to bypass or counteract this pathway to maintain a lipid-rich environment favorable for their survival [17,26].

Although AMPK signaling, fatty acid metabolism, and LD dynamics have been explored individually in various viral systems, their coordinated interplay during ZIKV infection, particularly in TM cells, remains poorly understood. We previously demonstrated that ZIKV infects TM cells and induces a dysregulated immune response [4,6]. Given the essential role of TM in regulating aqueous humor outflow and maintaining IOP, disruption of metabolic homeostasis in these cells could directly contribute to ocular disease progression. Therefore, elucidating how ZIKV alters energy and lipid metabolism in TM cells may provide critical mechanistic insights into disease pathogenesis and uncover potential therapeutic targets.

In this study, we examined how ZIKV reprograms host metabolic pathways in TM cells, with a particular focus on AMPK signaling, fatty acid metabolism, and LD biogenesis. Using primary human TM cells and an IFNAR1-deficient mouse model, we show that ZIKV differentially modulates these interconnected pathways to promote its replication and spread. Collectively, our findings provide new insights into the metabolic underpinnings of ZIKV-associated ocular disease and highlight host metabolic pathways as potential targets for therapeutic interventions.

## 2. Materials and Methods

### 2.1. Antibodies and Reagents

Antibodies used in this study were obtained from the following sources: 4G2 (GeneTex, #GTX57154), ZIKV NS3 (GeneTex, #GTX133309), and β-actin (Millipore Sigma, St. Louis, MO, USA) #A2228). Antibodies against pLKB1 (Thr189) (#3054S), LKB1 (#3050S) pAMPKα (Thr172) (#2535S), AMPKα (#5831S), pACC (Ser79) (#3661S), ACC (#3662S) were purchased from Cell Signaling Technology (Danvers, MA, USA). Antibodies against FASN (#10624-2-AP), CPT-1A (#15184-1-AP), and GIMAP2 (#13445-1-AP) were obtained from Proteintech (Rosemont, IL, USA), whereas Perilipin-3/TIP47 (#sc-390968), DGAT1 (#sc-271934), CD36 (#sc-7309), SREBP-1 (#sc-13551), and PPAR-γ (#sc-7273) were purchased from SantaCruz Biotechnology (Dallas, TX, USA). AICAR (#10010241), metformin (#13118), 2DG (#14325), dorsomorphin (#11967), etomoxir (#11969), and A922500 (#10012708) were purchased from Cayman Chemicals (Ann Arbor, MI, USA). Oleic acid (#O1257), linoleic acid (#L5900), and palmitic acid (#W283207) were obtained from Millipore Sigma (St. Louis, MO, USA).

### 2.2. Cell Culture

Normal primary human trabecular meshwork cells (HTMC) [4,6,27] were cultured in Dulbecco’s Modified Eagle medium (DMEM) low-glucose GlutaMAX supplemented with 10% fetal bovine serum (FBS), 1X penicillin–streptomycin (P/S), and maintained at 37 °C in a humidified CO_2_ (5%) incubator. Vero E6 cells (ATCC CRL-1586) were cultured in DMEM low-glucose GlutaMAX medium supplemented with 10% FBS and 1X penicillin–streptomycin under the same conditions. For virus propagation, *Aedes albopictus* C6/36 cells (ATCC CRL-1660) were maintained in Eagle’s Minimum Essential Medium (EMEM) supplemented with 10% FBS and 1X penicillin–streptomycin at 30 °C in a humidified incubator.

### 2.3. Zika Virus Propagation and Infection Procedure

The ZIKV strain PRVABC59 (NR-50240) was obtained from BEI Resources, National Institute of Allergy and Infectious Diseases (NIAID), and propagated in *Aedes albopictus*, C6/36 cells. Viral titers were determined by plaque assay. Virus stocks were aliquoted and stored at −80 °C until use.

For in vitro experiments, HTMCs were infected with ZIKV at a multiplicity of infection (MOI) of 1. For pharmacological modulation studies, cells were pretreated for 1 h with the indicated activators (AICAR, 1 mM; metformin, 20 mM; 2 DG, 10 µM), inhibitors (dorsomorphin, 10 µM; A922500, 10 µM; etomoxir, 50 µM), or fatty acids (linoleic acid, oleic acid, or palmitic acid, 250 µM), followed by virus adsorption in serum-free media for 1 h, unless otherwise specified. After adsorption, cells were replenished with complete medium containing the respective compounds and incubated until the indicated time points.

### 2.4. Mice and Ethics Statement

IFNAR1^−/−^ mice (C57BL/6 background, MMRRC Strain #032045-JAX) were originally obtained from Jackson Laboratories and bred in-house at the University of Missouri (MU) Office of Animal Resources (OAR) facility. Male and female mice aged 8–10 weeks were used for all experiments. Animals were housed in a restricted-access AAALAC-accredited facility under a 12:12 h light/ dark cycle, with ad libitum access to food (rodent chow, Labdiet Pico Laboratory, St. Louis, MO, USA) and water. All animal procedures were conducted in accordance with the Association for Research in Vision and Ophthalmology (ARVO) Statement for the Use of Animals in Ophthalmic and Vision Research. All biohazard and animal protocols were approved by the MU Institutional Biosafety Committee (IBC) and Animal Care and Use Committee (ACUC) under protocol #53402, approved on 23 July 2024.

### 2.5. Mouse Infection and Drug Treatment

For in vivo studies, mice were pretreated with the indicated drugs via i.p. injections starting one day prior to ZIKV infection and continuing for four consecutive days: AICAR, 100 mg/kg [28]; dorsomorphin, 10 mg/kg [29]; A922500, 3 mg/kg [30]; etomoxir, 15 mg/kg [31]; linoleic acid, 50 mg/kg [32]; or palmitic acid, 10 mg/kg [33]. For ZIKV infection, anesthetized mice were inoculated with 1 × 10^4^ PFU of ZIKV via intracameral injection, as we described previously [6,27]. At 7 days post-infection, fundus imaging and OCT were performed using a Micron IV retinal imaging system and an image-guided OCT system (Phoenix-Micron Inc., Bend, OR, USA). Mice were then euthanized, and the anterior segment tissue was collected for analysis of innate antiviral and inflammatory mediators by qPCR.

### 2.6. Viral Attachment, Entry, and Inactivation Assay

The viral attachment and entry assay were performed as we described previously [27,34]. Briefly, HTMCs were pre-incubated with oleic acid, linoleic acid, or palmitic acid (250 µM) for 1 h at 4 °C (attachment assay) or 37 °C (entry assay), followed by infection with ZIKV at an MOI of 1. After 2 h of viral adsorption, cells were washed three times with fresh DMEM to remove unbound virus, and total RNA was isolated. Viral RNA copy numbers were quantified from whole-cell RNA using a TaqMan probe targeting the ZIKV envelope (E) gene by qPCR.

For the direct viral inactivation assay, ZIKV (1 × 10^6^ PFU/mL) was incubated with oleic acid, linoleic acid, or palmitic acid in a cell-free medium (serum-free DMEM) at 37 °C for 2 or 4 h. ZIKV incubated without any compounds served as untreated control. Following incubation, 100 µL of each virus-fatty acid mixture was serially diluted (10^−2^ to 10^−5^ fold), and viral infectivity was determined by plaque assay.

### 2.7. Plaque Assay

The viral titers were determined by plaque assay using a protocol we described recently [4,27]. Briefly, confluent monolayers of Vero cells were infected with serial dilutions of treated or untreated conditioned media. After 1h of viral adsorption, the cell monolayer was overlaid with the first overlay media containing a 1:1 mixture of 2X DMEM supplemented with 4% FBS, 2X P/S, 20 mM MgCl_2_, and 1.6% Noble agar. On the following day, a second overlay medium containing DMEM supplemented with 1 mg/mL BSA, 40 mM MgCl_2_, 0.2% glucose, 2 mM sodium pyruvate, 4 mM L-glutamine, 4 mM oxaloacetic acid, 1X P/S, and 0.1% sodium bicarbonate was added. Plates were incubated at 37 °C in a CO_2_ incubator for 5 days. Following incubation, cells were fixed with 10% tricarboxylic acid (TCA) for 20 min, and the agar overlay was carefully removed without disturbing the cell monolayer. Viral plaques were visualized by staining with 0.2% crystal violet for 20 min, followed by washing with Milli-Q water. Plaques were counted, and viral titers were calculated as log_10_ PFU/mL.

### 2.8. Immunofluorescence Staining

For immunofluorescence analysis, HTMCs were seeded in Nunc four-well chamber slides (Fisher Scientific, Rochester, NY, USA) and infected with ZIKV at an MOI of 1 at ~70–80% confluency. Mock-treated/ uninfected cells served as controls. At the indicated time points, cells were fixed with 4% paraformaldehyde for 10 min at room temperature (RT). Following three washes with 1X PBS, cells were blocked and permeabilized with 1% (*w*/*v*) BSA with 0.4% (*v*/*v*) Triton X-100 made in PBS (blocking buffer) for 1 h at RT in a humidified chamber. Cells were then incubated with the primary mouse or rabbit antibodies diluted (1:100 dilutions) in the blocking buffer overnight at 4 °C in a humidified chamber. After three washes with 1X PBS, cells were incubated with Alexa Fluor 488- or 594-conjugated anti-mouse or anti-rabbit secondary antibodies (1:200 dilutions) for 1 h at RT. Finally, cells were washed four times with 1X PBS and mounted using Vectashield antifade mounting medium containing DAPI (Vector Laboratories, Burlingame, CA, USA). The slides were visualized and imaged using a Keyence fluorescence microscope (Keyence, Itasca, IL, USA).

### 2.9. Lipid Droplet Staining

HTMCs were seeded in Nunc four-well chamber slides (Fisher Scientific, Rochester, NY, USA) and infected with ZIKV at an MOI of 1 for the indicated timepoints. Mock-treated/ uninfected cells served as controls. At the indicated time points, cells were fixed with 4% paraformaldehyde and stained for lipid droplets using the HSC LipidTOX™ Green Neutral Lipid Stain Kit (#H34475) according to the manufacturer’s instructions (ThermoFisher Scientific, Rockford, IL, USA). The slides were visualized and imaged using a Keyence fluorescence microscope (Keyence, Itasca, IL, USA).

### 2.10. Western Blotting

Western blotting was performed using a method we described previously [4,27]. Briefly, treated and untreated cells were washed with ice-cold PBS and lysed in RIPA buffer containing Halt^TM^ protease and phosphatase inhibitor cocktail (ThermoFisher Scientific, Rockford, IL, USA). Total protein concentration was determined using a BCA protein assay kit according to the manufacturer’s instructions (ThermoFisher Scientific, Rockford, IL, USA). Equal amounts of protein (30 µg) were resolved by SDS-PAGE and transferred onto nitrocellulose or PVDF membranes. Following transfer, membranes were blocked in 5% non-fat skim milk prepared in 1X TBST. Membranes were then incubated with anti-rabbit or anti-mouse primary antibodies (1:1000 dilutions) diluted in 3% BSA overnight at 4 °C with gentle agitation. After primary antibody incubation, membranes were washed three times with 1X TBST and incubated with HRP-conjugated anti-mouse or anti-rabbit secondary antibodies (1:2000 dilutions) for 2 h at RT. After three additional washes with 1X TBST, protein bands were detected using SuperSignal West Femto chemiluminescent substrate and imaged using an iBright FL1500 imaging system (Thermo Fisher Scientific, Rockford, IL, USA).

### 2.11. RNA Isolation and qRT-PCR

Following treatment, cells were harvested in TRIzol™ reagent (#15596018), and total RNA was isolated according to the manufacturer’s instructions (ThermoFisher Scientific, Rockford, IL, USA). One microgram of RNA was reverse-transcribed into cDNA using the Maxima First-Strand cDNA Synthesis Kit, following the manufacturer’s protocol (ThermoFisher Scientific, Rockford, IL, USA). qRT-PCR was performed using human or mouse gene-specific primers in a 96-well plate on a QuantStudio 3 Real-Time PCR system (ThermoFisher Scientific, Rockford, IL, USA). Relative mRNA expression levels were normalized to the housekeeping gene 18S rRNA. Data were analyzed using the 2^−ΔΔCT^ method and are presented as relative fold change.

### 2.12. Statistical Analysis

Statistical analyses were performed using GraphPad Prism v10.1.2 (GraphPad Software, La Jolla, CA, USA). A one-way or two-way ANOVA was used to assess statistical differences among experimental groups. A *p*-value < 0.05 was considered statistically significant. Data are presented as mean ± SD from three independent biological replicates, unless indicated otherwise.

## 3. Results

### 3.1. ZIKV Activates the LKB1-AMPK-ACC Signaling Axis in HTMCs

**Figure 1:**
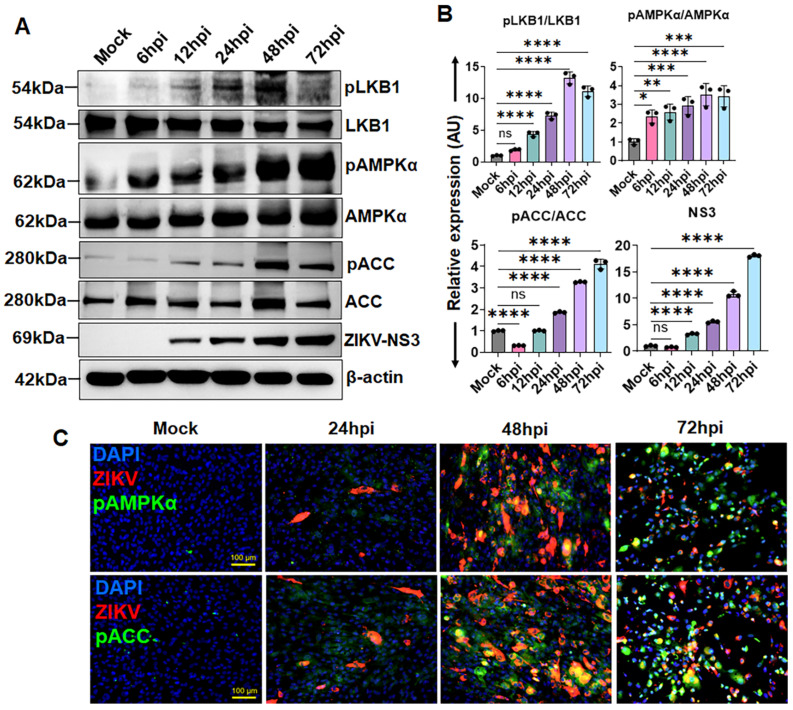
**ZIKV activates the LKB1-AMPK-ACC signaling axis in HTMCs.** HTMCs (n = 3) were infected with ZIKV for the indicated time points. Mock-treated cells were used as controls. (**A**) Infected and mock-treated cells were analyzed by immunoblotting for phosphorylated and total LKB1, AMPKα, and ACC. Immunoblotting for ZIKV non-structural protein NS3 was used as an infection control. (**B**) Densitometric analysis of immunoblots was performed using ImageJ 1.54g, and protein expression was normalized to β-actin. (**C**) In a second set of experiments, infected and mock-treated cells were fixed and immunostained with antibodies against pAMPKα, pACC, and the flavivirus envelope (E) antigen (4G2). Representative images show phospho-proteins (Green), ZIKV (Red), and DAPI-stained nuclei (Blue); scale bar: 100 µm. Bar graphs represent mean ± SD from three biological replicates. * *p* < 0.05; ** *p* < 0.005; *** *p* < 0.0005; **** *p* < 0.0001; ns = not significant (one-way ANOVA).

AMPK is a central regulator of cellular energy homeostasis and plays a critical role in coordinating metabolic responses during viral infection. Several viruses modulate AMPK activity to support their replication and persistence [17,18]. We previously observed that ZIKV differentially regulates AMPK in ocular cells, suppressing its activity in retinal vascular endothelial cells [18], while activating it in retinal pigmented epithelial cells (unpublished data). However, how ZIKV modulates AMPK signaling in the anterior segment, particularly in TM cells, remains unclear. To address this, we examined AMPK activity in primary human TM cells (HTMCs) by assessing the phosphorylation status of the LKB1-AMPK-ACC signaling axis over time. Our immunoblot analysis showed that ZIKV infection induced a time-dependent increase in phosphorylation of LKB1, along with its downstream mediators, AMPKα (Thr172) and ACC (Ser79) (Figure 1A,B). To further validate these findings, we performed immunofluorescence staining for phosphorylated AMPKα and ACC. Consistent with the immunoblot data, ZIKV-infected HTMCs exhibited a progressive increase in pAMPKα and pACC signal intensity over time compared to mock controls (Figure 1C). ZIKV infectivity was confirmed by NS3 immunoblotting and 4G2 immunostaining in these experiments (Figure 1A–C).

### 3.2. AMPK Activation Restricts, Whereas Its Inhibition Enhances, ZIKV Replication in HTMCs

**Figure 2:**
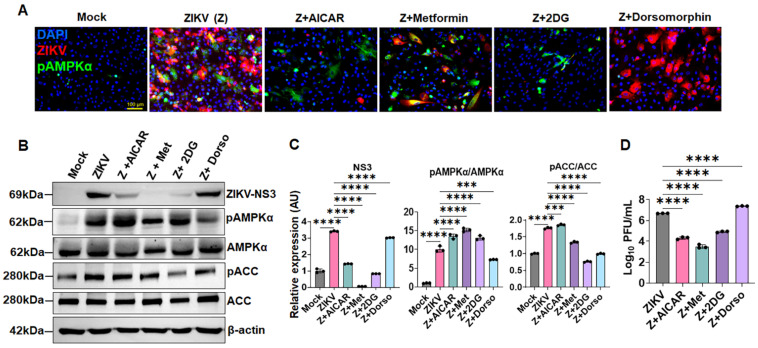
**AMPK activation restricts, whereas its inhibition enhances, ZIKV replication in HTMCs.** HTMCs (n = 3) were pretreated with AMPK activators (AICAR, metformin, 2DG) or the inhibitor dorsomorphin, followed by infection with ZIKV (Z) for 48 h. Mock-treated cells served as controls. (**A**) Cells were fixed and immunostained for 4G2 and pAMPKα antibodies. Representative images show the presence of ZIKV (Red), pAMPKα (Green), and DAPI-stained nuclei (Blue); scale bar: 100 µm. (**B**) In a parallel experiment, cell lysates from treated and ZIKV-infected cells were analyzed by immunoblotting for ZIKV NS3, along with pAMPKα, AMPKα, pACC, ACC, and β-actin. (**C**) Densitometric analysis of immunoblots was performed using ImageJ 1.54g, and protein expression was normalized to β-actin. (**D**) Conditioned media from treated and untreated cells were subjected to plaque assay to quantify the replicating virions. The plaques were counted and expressed as Log_10_ PFU/mL. Bar graphs represent mean ± SD from three biological replicates. *** *p* < 0.0005; **** *p* < 0.0001; (one-way ANOVA).

Given that ZIKV activates AMPK signaling in HTMCs, we next examined whether modulation of AMPK activity directly influences viral replication. To test this, we pretreated primary HTMCs with the AMPK activators—AICAR, metformin, and 2-Deoxy-D-glucose (2DG)—prior to ZIKV infection. Consistent with our previous findings [4,6], ZIKV permissively infected untreated HTMCs; whereas treatment with AMPK activators markedly reduced viral replication as evidenced by a significant decrease in immunofluorescence staining for ZIKV envelope (E) antigen 4G2 (Figure 2A). In contrast, AMPK inhibition using dorsomorphin resulted in increased 4G2 staining, indicating enhanced viral replication (Figure 2A).

To further validate these findings, we assessed viral replication by immunoblotting for the ZIKV non-structural protein NS3 and by measuring infectious virion production using plaque assays. Consistent with the immunofluorescence results, treatment with AICAR, metformin, or 2DG significantly reduced ZIKV NS3 protein expression, along with a corresponding increase in phosphorylated AMPKα and ACC (Figure 2B,C). Conversely, dorsomorphin-mediated inhibition of AMPK led to a marked increase in NS3 expression along with decreased phosphorylation of AMPKα and ACC (Figure 2B,C), further supporting enhanced viral replication under conditions of AMPK suppression. Our plaque assays further confirmed that AMPK activation significantly suppressed infectious virus production, as evidenced by a significant decrease in the number of plaques with AMPK activators, AICAR, metformin, and 2DG treatments (Figure 2D). Together, these findings demonstrate that AMPK activation restricts ZIKV replication, while its inhibition enhances viral propagation in HTMCs.

### 3.3. AMPK Activation Enhances Antiviral Responses While Limiting ZIKV-Induced Inflammation in HTMCs

**Figure 3:**
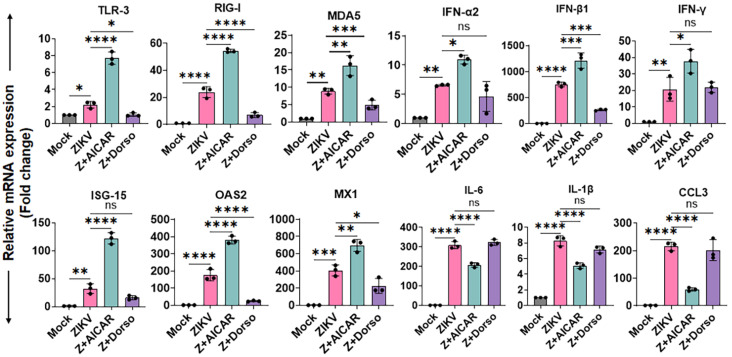
**AMPK activation enhances antiviral responses and limits ZIKV-induced inflammation in HTMCs.** HTMCs (n = 3) were pretreated with AMPK activators AICAR or the inhibitor dorsomorphin, followed by infection with ZIKV (Z) for 48 h. Mock-treated cells served as controls. Total RNA was extracted and subjected to qPCR to quantify the mRNA expression of the indicated genes. Gene expression levels were normalized to 18S rRNA and expressed as relative fold changes. Bar graphs represent mean ± SD from three biological replicates. * *p* < 0.05; ** *p* < 0.005; *** *p* < 0.0005; **** *p* < 0.0001; ns = not significant (one-way ANOVA).

In addition to regulating metabolism, AMPK is an important modulator of innate antiviral immunity [15,17,18]. We observed that ZIKV activates AMPK, likely as part of a metabolic adaptation to increased energy demands during infection; however, AMPK activators paradoxically limit viral replication. This prompted us to investigate how AMPK influences host antiviral and inflammatory responses during infection. To address this, we assessed the expression of key components of viral immunity, including pattern recognition receptors (PRRs), interferons (IFNs), interferon-stimulated genes (ISGs), and pro-inflammatory mediators. We pretreated HTMCs with the AMPK activator AICAR or the inhibitor dorsomorphin prior to ZIKV infection, and quantified gene expression by qPCR. Our results show that AMPK activation significantly enhanced the antiviral response, as evidenced by increased expression of PRRs, including TLR-3, RIG-I, and MDA5. This was accompanied by a marked upregulation of type I and II IFNs (IFN-α2, IFN-β1, IFN-γ), along with downstream ISGs, such as ISG-15, OAS2, and MX1 (Figure 3). In contrast, AMPK inhibition suppressed the expression of these antiviral genes, indicating that AMPK activation promotes a robust antiviral immune response to ZIKV infection. In addition to promoting antiviral signaling, AMPK activation attenuated the ZIKV-induced pro-inflammatory response. AICAR treatment significantly reduced the expression of inflammatory cytokines and chemokines, such as IL-6, IL-1β, and CCL3, compared to untreated ZIKV-infected and dorsomorphin-treated cells (Figure 3). Together, these findings demonstrate that AMPK activation not only promotes antiviral immunity but also helps limit inflammatory responses during ZIKV infection in HTMCs.

### 3.4. ZIKV Reprograms Lipid Metabolism and Induces LD Biogenesis in HTMCs

**Figure 4:**
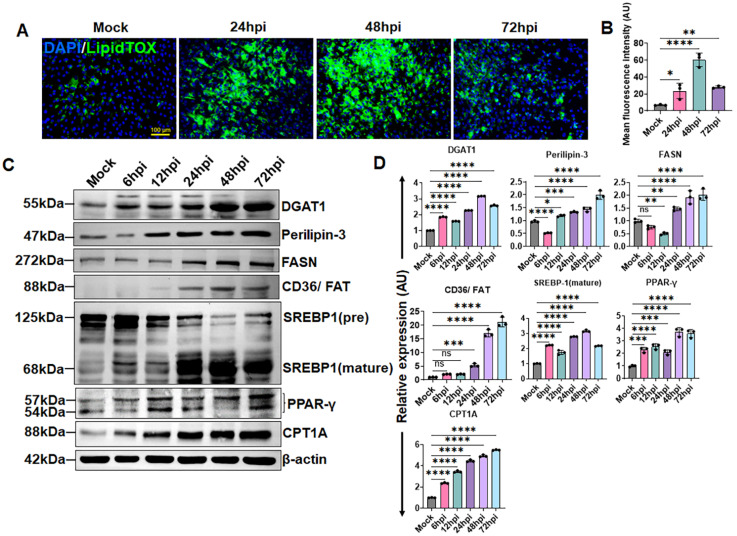
**ZIKV enhances LD biogenesis by reprogramming lipid metabolism in HTMCs.** HTMCs (n = 3) were infected with ZIKV for the indicated time points. Mock-treated cells were used as controls. (**A**) Cells were briefly fixed and stained for LD using LipidTOX Green. Representative images show LDs (Green) and DAPI-stained nuclei (Blue); scale bar: 100 µm. (**B**) The LD fluorescence intensity was quantified using ImageJ. (**C**) In parallel experiments, infected and mock-treated cells were analyzed by immunoblotting for lipid metabolism-associated proteins: DGAT1, perilipin-3, FASN, CD36/FAT, SREBP1, PPAR-γ, CPT1A, and the housekeeping protein β-actin. (**D**) Densitometric analysis of immunoblots was performed using ImageJ 1.54g, and protein expression was normalized to β-actin. Bar graphs represent mean ± SD from three biological replicates. * *p* < 0.05; ** *p* < 0.005; *** *p* < 0.0005; **** *p* < 0.0001; ns = not significant (one-way ANOVA).

Flaviviruses are known to manipulate host lipid metabolism to support viral replication and assembly [35,36,37]. LDs are dynamic organelles that function as energy reservoirs and serve as platforms for viral replication. Many viruses trigger LD formation to meet their lipid and energy demands, making LD accumulation one of the most prominent cellular changes following infection. LDs are generated when diacylglycerol (DAG) is acylated by diacylglycerol acyltransferases such as DGAT1, linking their formation directly to cellular lipid metabolism [38]. Previous studies have suggested that ZIKV modulates fatty acid and lipid metabolism to support its replication in neural stem cells [36] and retinal pigmented epithelial cells [35]. In our study, because we observed ZIKV-induced AMPK activation, which typically suppresses lipid synthesis, we initially hypothesized that ZIKV infection might suppress host lipid biosynthetic pathways. To investigate how ZIKV affects host lipid metabolism in HTMCs, first, we measure LD accumulation over time using LipidTOX Green, a lipid stain with high affinity for neutral lipid droplets. Contrary to our expectations, ZIKV infection led to a robust, time-dependent increase in LD formation compared with mock-treated HTMCs (Figure 4A,B).

To further explore the molecular basis of this LD accumulation, we assessed the expression of key lipid-regulatory proteins. Interestingly, ZIKV infection increased the levels of DGAT1, perilipin-3, fatty acid synthase (FASN), and CD36/FAT relative to uninfected controls (Figure 4C,D). Additionally, HTMC infected with ZIKV showed increased expression of the fatty acids β-oxidation enzyme CPT1A and the lipogenic transcription factor peroxisome proliferator-activated receptor-γ (PPAR-γ) in a time-dependent manner (Figure 4C,D). Notably, ZIKV also promoted the induction of sterol regulatory element-binding protein 1 (SREBP-1) to its mature, transcriptionally active form, indicating enhanced lipogenic signaling (Figure 4C,D). Together, these findings indicate that ZIKV drives a coordinated reprogramming of host lipid metabolism, enhancing lipogenesis, lipid storage, and fatty acid oxidation, to promote lipid droplet biogenesis and generate a lipid-rich environment that supports viral replication, despite concurrent AMPK activation.

### 3.5. Inhibition of LD Biogenesis and β-Oxidation Reduces ZIKV Replication in HTMCs

Viruses rely on host lipid metabolism for both membrane synthesis and energy production during replication. DGAT1 catalyzes the final step in triacylglycerol synthesis and is essential for LD formation, whereas CPT1A regulates mitochondrial fatty acid β-oxidation and ATP generation. Previous studies have shown that targeting DGAT1 can limit replication of several viruses, including ZIKV, DENV, hepatitis C virus, and SARS-CoV-2 [36,37,39,40,41], while inhibition of CPT1A impairs DENV replication [42].

**Figure 5:**
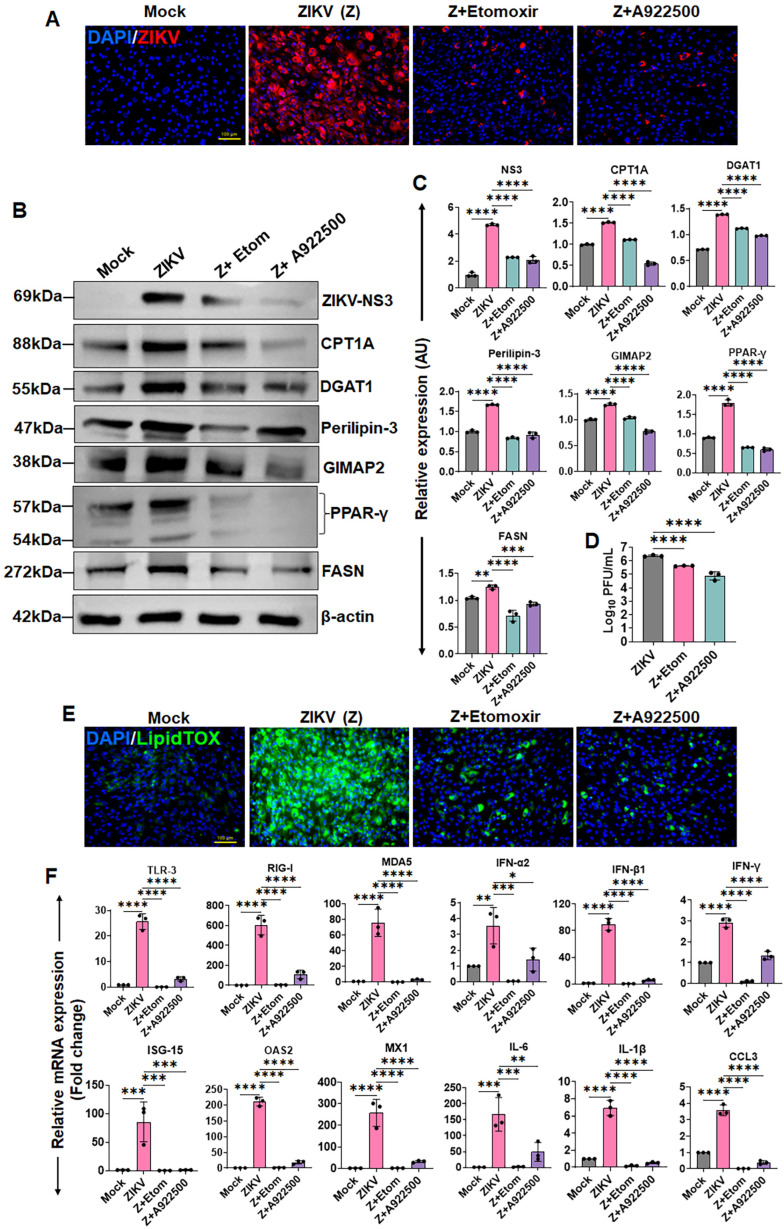
**Inhibition of DGAT1 (LD biogenesis) and CPT1A (β-oxidation) reduces ZIKV replication in HTMCs.** HTMCs (n = 3) were pretreated with the DGAT1 inhibitor A922500 or the CPT1A inhibitor etomoxir, followed by infection with ZIKV (Z) for 48 h. Mock-treated cells served as controls. (**A**) Cells were fixed and immunostained for 4G2 antibodies. Representative images show ZIKV (Red) and DAPI-stained nuclei (Blue); scale bar: 100 µm. (**B**) In parallel, cell lysates from treated and ZIKV-infected cells were analyzed by immunoblotting for ZIKV NS3, and lipid metabolism-associated proteins, including DGAT1, perillpin-3, GIMAP2, PPAR-γ, FASN, CPT1A, and the housekeeping protein β-actin. (**C**) Densitometric analysis of immunoblots was performed using ImageJ 1.54g, and protein expression was normalized to β-actin. (**D**) Conditioned media from treated and untreated cells were subjected to plaque assay to quantify the replicating virions. The plaques were counted and expressed as Log_10_ PFU/mL. (**E**) Treated and untreated cells were stained for LD using LipidTOX Green. Representative images show LDs (Green) and DAPI-stained nuclei (Blue); scale bar: 100 µm. (**F**) In parallel experiments, total RNA was extracted and subjected to qPCR to quantify mRNA expression of the indicated genes. Gene expression levels were normalized to 18S rRNA and expressed as relative fold changes. Bar graphs represent mean ± SD from three biological replicates. * *p* < 0.05; ** *p* < 0.005; *** *p* < 0.0005; **** *p* < 0.0001; ns = not significant (one-way ANOVA).

Our aforementioned results indicated that ZIKV infection upregulates both DGAT1 and CPT1A, suggesting a coordinated enhancement of lipid synthesis and fatty acid oxidation. We therefore asked whether pharmacological inhibition of these pathways would impact ZIKV replication in HTMCs. To test this, we pretreated cells with DGAT1 inhibitor A922500 or CPT1A inhibitor etomoxir prior to viral infection. Following treatment, viral replication was assessed by immunofluorescence staining for the ZIKV envelope antigen (4G2), immunoblotting for ZIKV non-structural protein NS3, and quantification of infectious virions using plaque assay. Our results revealed that inhibition of either DGAT1 or CPT1A significantly reduced ZIKV replication, as evidenced by decreased 4G2 staining (Figure 5A), reduced NS3 protein expression (Figure 5B,C), and a marked decline in plaque formation (Figure 5D). Immunoblot analysis confirmed that treatment with these inhibitors suppressed the expression of key lipid metabolic regulators, including CPT1A, DGAT1, perilipin-3, GIMAP2, PPAR-γ, and FASN (Figure 5B,C), validating effective pathway inhibition. The inhibition of LD biogenesis by A922500 and etomoxir was confirmed by LipidTOX staining, which shows significantly reduced LD accumulation compared to ZIKV-infected cells (Figure 5E).

To further assess the impact of lipid metabolic disruption on host antiviral response, we measured the expression of key immune genes. Our results show that treatment with A922500 or etomoxir significantly reduced the expression of PRRs (TLR-3, RIG-I, MDA5), IFNs (IFN-α2, IFN-β1, IFN-γ), and ISGs (ISG-15, OAS2, MX1), as well as pro-inflammatory mediators (IL-6, IL-1β, and CCL3) (Figure 5F). This decrease is likely attributed to the reduced viral burden following inhibition of lipid metabolic pathways. Collectively, these findings demonstrate that both LD biogenesis and fatty acid β-oxidation are critical for efficient ZIKV replication in HTMCs, and that disruption of these pathways significantly impairs viral propagation.

### 3.6. Fatty Acid Supplementation Differentially Modulates ZIKV Replication in HTMCs

Because inhibition of both LD biogenesis and fatty acid β-oxidation significantly reduced ZIKV replication in HTMCs, we next investigated whether increasing lipid availability could promote ZIKV replication. Fatty acids such as linoleic acid, oleic acid, and palmitic acid are known to increase LD accumulation and alter cellular lipid metabolism [43,44,45].

**Figure 6:**
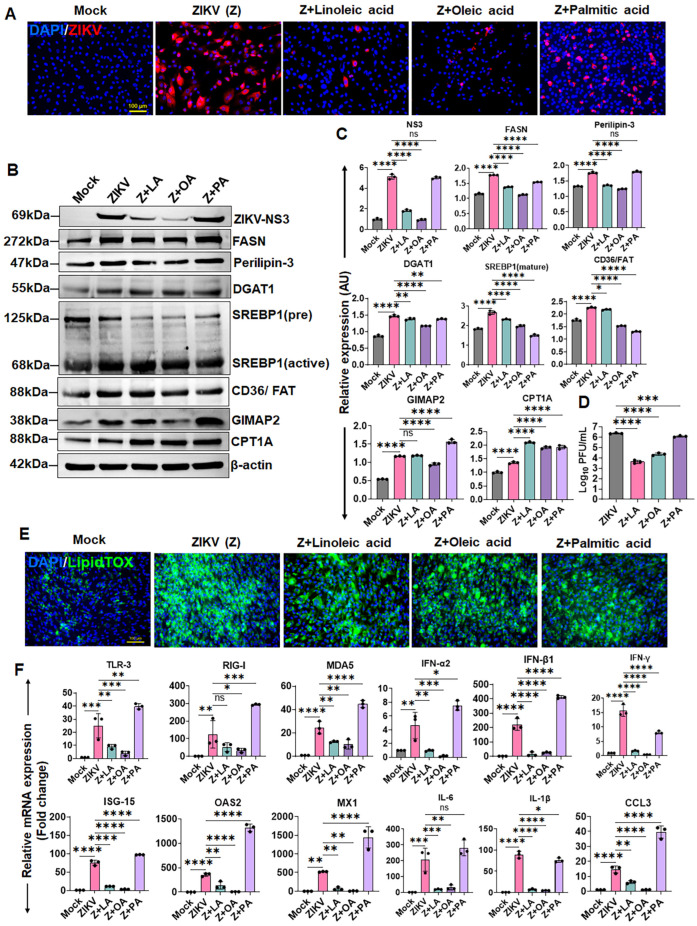
**Unsaturated and saturated fatty acids differentially modulate ZIKV replication in HTMCs.** HTMCs (n = 3) were pretreated with LA, OA, or PA, followed by infection with ZIKV (Z) for 48 h. Mock-treated cells served as controls. (**A**) Cells were fixed and immunostained for 4G2 antibodies. Representative images show ZIKV (Red) and DAPI-stained nuclei (Blue); scale bar: 100 µm. (**B**) In parallel, cell lysates from treated and ZIKV-infected cells were analyzed by immunoblotting for ZIKV NS3, and lipid metabolism-associated proteins, including FASN, perillpin-3, DGAT1, SREBP1, CD36/FAT, GIMAP2, CPT1A, and the housekeeping protein β-actin. (**C**) Densitometric analysis of immunoblots was performed using ImageJ 1.54g, and protein expression was normalized to β-actin. (**D**) Conditioned media were subjected to plaque assay to quantify the infectious virions. The plaques were counted and expressed as Log_10_ PFU/mL. (**E**) Cells were stained for neutral lipids using LipidTOX Green. Representative images show LDs (Green) and DAPI-stained nuclei (Blue); scale bar: 100 µm. (**F**) Total RNA was extracted and subjected to qPCR to quantify mRNA expression of the indicated genes. Bar graphs represent mean ± SD from three biological replicates. * *p* < 0.05; ** *p* < 0.005; *** *p* < 0.0005; **** *p* < 0.0001; ns = not significant (one-way ANOVA).

To examine their effects on ZIKV, we pretreated HTMCs with linoleic acid, oleic acid, or palmitic acid and assessed viral replication by immunofluorescence staining for the viral E antigen (4G2), immunoblotting for the ZIKV NS3 protein, and plaque assays to quantify infectious virions. Interestingly, despite all treatments increasing LD accumulation, their effects on ZIKV replication differed. Treatment with the unsaturated fatty acids, linoleic acid and oleic acid, significantly reduced viral replication, as evidenced by decreased 4G2 staining (Figure 6A), reduced NS3 expression (Figure 6B,C), and a decreased number of plaques (Figure 6D). In contrast, the saturated fatty acid palmitic acid either enhanced or had no significant effect on viral replication compared to ZIKV-infected untreated controls (Figure 6A–D). The effects of fatty acid supplementation on LD biogenesis were confirmed by LipidTOX staining, which demonstrated increased LD accumulation (Figure 6E), and by immunoblot analysis of key lipid metabolic regulators, including FASN, perilipin-3, DGAT1, SREBP1, CD36/FAT, GIMAP2, and CPT1A (Figure 6B,C).

To further determine how these fatty acid treatments influence host immune responses, we assessed the expression of key antiviral and inflammatory genes. Consistent with reduced viral burden, unsaturated fatty acids (linoleic and oleic acids) significantly reduced the expression of PRRs (TLR-3, RIG-I, MDA5), type I and II IFNs (IFN-α2, IFN-β1, IFN-γ), and ISGs (ISG-15, OAS2, MX1), as well as inflammatory mediators (IL-6, IL-1β, and CCL3) (Figure 6F). In contrast, palmitic acid (saturated fatty acid) either enhanced or had a comparable effect on the expression of these genes with respect to untreated ZIKV-infected cells. Collectively, these findings indicate that fatty acid supplementation differentially regulates ZIKV replication and associated host responses. Fatty acid composition, rather than total lipid abundance, determines the outcome of ZIKV infection in HTMCs.

### 3.7. Unsaturated Fatty Acids Inhibit ZIKV by Targeting Early Stages of Infection and Directly Inactivating Virions

**Figure 7:**
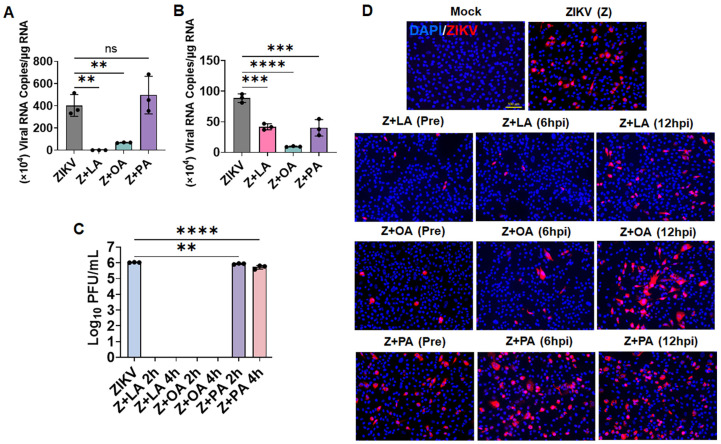
**Unsaturated fatty acids inhibit ZIKV infection by direct viral inactivation and by preventing viral binding and entry into cells.** (**A**) Viral attachment assay showing ZIKV E gene copies quantified by qPCR. Data are presented as mean ± SD of RNA copies/μg RNA. (**B**) Viral entry assay showing ZIKV E gene copies quantified by qPCR. Data are shown as mean ± SD of RNA copies/μg RNA. (**C**) Viral inactivation assay was performed in the presence or absence of FAs, and viral progeny were quantified by plaque assay. The plaque counts were expressed as Log_10_ PFU/mL. (**D**) HTMC were either pretreated (1 h before infection) with LA, OA, or PA, or treated 6 h or 12 h post-ZIKV infection. Mock-treated and ZIKV-infected cells without treatment served as controls. Forty-eight hours post-infection, cells were fixed and immunostained for ZIKV E antigen (4G2). Representative images show ZIKV (Red) and DAPI-stained nuclei (Blue), scale bar: 100 µm. ** *p* < 0.005; *** *p* <  0.0005; **** *p* < 0.0001; ns = not significant (one-way ANOVA).

We were puzzled by our observation that, despite increased LD biogenesis induced by these fatty acids, ZIKV replication differed across treatments. Given this divergence, despite all treatments promoting LD accumulation, we next sought to define the underlying mechanisms. Based on our previous findings that short-chain fatty acids can directly inactivate ZIKV and interfere with viral entry [27], we investigated whether these long-chain saturated and unsaturated fatty acids similarly affect early stages of the viral life cycle. To assess viral attachment and entry, we treated HTMCs with linoleic acid, oleic acid, or palmitic acid, followed by quantification of viral RNA copy numbers under conditions that selectively assess binding or internalization [27,34]. In the viral attachment assay, treatment with the unsaturated fatty acids linoleic acid and oleic acid significantly reduced viral RNA copy numbers compared to untreated infected controls, indicating impaired viral binding (Figure 7A). In contrast, the saturated fatty acid palmitic acid had no significant effect on viral attachment. In the viral entry assay, all three fatty acids reduced viral RNA copy numbers to varying degrees, suggesting that fatty acids can interfere with viral internalization (Figure 7B). However, the inhibitory effect was more pronounced with linoleic and oleic acids. To determine whether these fatty acids exert direct virucidal activity, ZIKV was incubated with these fatty acids in a cell-free medium for 2 and 4 h. Following incubation, the fatty acid–viral mixtures were serially diluted (2–5 log), and the viral infectivity was quantified by plaque assay. Our plaque assays revealed that linoleic acid and oleic acid completely abolished plaque formation, indicating direct viral inactivation (Figure 7C). In contrast, palmitic acid exhibits minimal or no virucidal activity under the same conditions.

We further evaluated the stage-specific effects of these fatty acids using pre- and post-infection treatment approaches. Pretreatment with linoleic and oleic acids significantly reduced viral replication, consistent with their ability to impair viral attachment and entry. Although post-infection treatment (6 and 12 hpi) also reduced viral replication to some extent, the inhibitory effect was less pronounced compared to pretreatment (Figure 7D). In contrast, palmitic acid showed minimal to no effect under either pre- or post-treatment conditions (Figure 7D). Collectively, these findings demonstrate that unsaturated fatty acids inhibit ZIKV primarily by targeting early stages of the viral life cycle, including viral attachment and entry, and by directly inactivating virions. In contrast, saturated fatty acids, such as palmitate, exhibit little to no antiviral activity under these conditions.

### 3.8. Modulation of Energy and Lipid Metabolism Alleviates ZIKV-Induced Ocular Pathology and Anterior Segment Inflammation in Mice

We previously demonstrated that ZIKV exhibits tropism for both anterior and posterior segments of the eye, leading to TM damage, chorioretinal atrophy, and retinal and optic nerve injury [4,5,6]. Building on our in vitro findings showing that ZIKV differentially modulates cellular energy (AMPK-ACC axis) and lipid metabolism (fatty acid synthesis and β-oxidation), we next investigated whether targeting these pathways could mitigate ZIKV-induced ocular disease in vivo.

To assess this, we utilized our previously established acute ZIKV infection model in IFNAR1-deficient mice [4,6,27]. We treated these mice with the AMPK activator AICAR, the AMPK inhibitor dorsomorphin, the DGAT1 inhibitor A922500, the CPT1A inhibitor etomoxir, or supplemented with unsaturated (linoleic) or saturated (palmitic) acids via i.p. administration. Mice were treated with these activators/inhibitors one day prior to ZIKV infection and continued for four consecutive days. Ocular pathology was assessed at 7 days post- infection using fundus imaging and optical coherence tomography (OCT).

**Figure 8:**
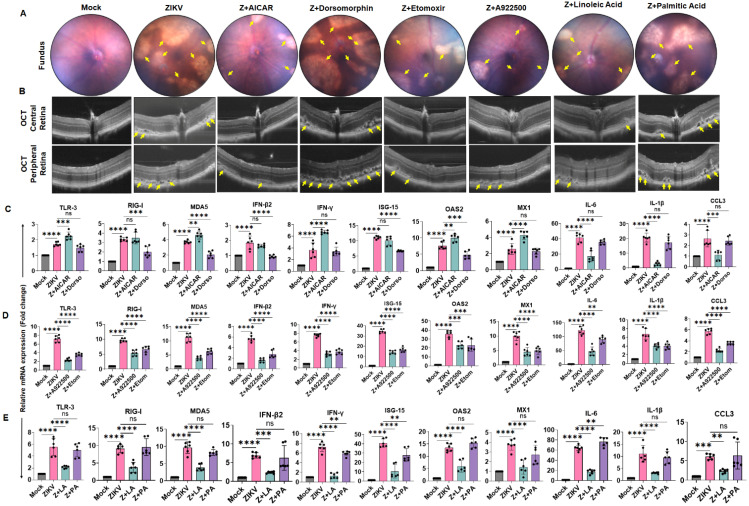
**Modulation of energy and lipid metabolism alleviates ZIKV-induced ocular pathology and anterior segment inflammation in mice.** IFNAR1^−/−^ mice (n = 6) were pretreated via i.p. injection with AMPK activator AICAR, AMPK inhibitor dorsomorphin, CPT1A inhibitor etomoxir, DGAT1 inhibitor A922500, unsaturated fatty acid linoleic acid, or saturated fatty acid palmitic acid from day −1 to 3 days post-infection (DPI). One day post-treatment, mice were infected with ZIKV (1 × 10^4^ PFU) via intracameral injection. Saline-injected mice without ZIKV infection served as mock controls. (**A**) Fundus and (**B**) OCT imaging was performed using the Micron IV fundus camera and image-guided OCT2 system to assess ocular pathology. Representative funduscopic images show RPE and chorioretinal atrophy (indicated with yellow arrows), and OCT images show RPE and outer retinal layer disruption (indicated with yellow arrows) in treated and untreated groups. (**C**–**E**) Total RNA was extracted from the anterior segment tissue of treated and untreated mice and analyzed by qPCR to quantify mRNA expression of the indicated genes. Bar graphs represent mean ± SD from six biological replicates. ** *p* < 0.005; *** *p* < 0.0005; **** *p* < 0.0001; ns = not significant (one-way ANOVA).

As expected, ZIKV infection resulted in pronounced retinal pathology, including RPE/chorioretinal atrophy and disruption of the outer retinal layers compared with uninfected controls (Figure 8A,B). In contrast, treatment with AICAR, A922500, etomoxir, or linoleic acid markedly attenuated ZIKV-induced retinal pathology, as evidenced by improved fundus appearance (Figure 8A) and preservation of retinal structure on OCT (Figure 8B). Notably, treatment with dorsomorphin or palmitic acid had minimal or no protective effect on retinal integrity (Figure 8A,B).

To further assess the impact of metabolic modulation on host immune responses, we analyzed the expression of innate antiviral and inflammatory genes in anterior segment tissue via qPCR. Consistent with our in vitro findings, AMPK activation with AICAR enhanced antiviral responses, whereas AMPK inhibition with dorsomorphin suppressed antiviral gene expression and exacerbated ZIKV-induced inflammation (Figure 8C). In contrast, inhibition of lipid metabolic pathways with DGAT1 inhibition (A922500) or CPT1A inhibition (etomoxir) significantly reduced the expression of ZIKV-induced PRRs (TLR-3, RIG-I, MDA5), IFNs (IFN-β2, IFN-γ), ISGs (ISG-15, OAS2, MX1), and inflammatory mediators (IL-6, IL-1β, and CCL3) (Figure 8D), consistent with decreased viral burden.

Finally, we evaluated the effects of fatty acid supplementation on host responses in vivo. Our data show that treatment with the unsaturated fatty acid linoleic acid significantly suppressed the expression of antiviral and inflammatory genes, whereas saturated fatty acid palmitic acid had minimal or no significant effect on these responses in comparison to ZIKV-infected and untreated mice (Figure 8E). Together, these findings demonstrate that targeting host energy and lipid metabolism, through AMPK activation, inhibition of lipid synthesis or β-oxidation, or supplementation with unsaturated fatty acids, can effectively reduce ZIKV-induced ocular pathology and associated inflammation in vivo.

## 4. Discussion

ZIKV infection during pregnancy is strongly associated with congenital abnormalities, including microcephaly and a spectrum of ocular manifestations such as chorioretinal atrophy, RPE disruption, optic nerve damage, and congenital glaucoma. Increasing evidence supports a strong ocular tropism of ZIKV; however, the mechanisms underlying anterior segment pathology, particularly TM dysfunction and elevated IOP, remain poorly understood. Given the critical role of TM in regulating aqueous humor outflow and maintaining IOP homeostasis, viral-mediated disruption of TM tissue represents a plausible driver of glaucomatous pathology. We previously demonstrated that ZIKV permissively infects anterior segment tissues, leading to TM damage, increased IOP, retinal ganglion cell loss, and inducing a dysregulated immune response [4,6]. However, the metabolic mechanisms underlying ZIKV replication in TM cells remain unclear.

In this study, we identify host energy and lipid metabolism as key regulators of ZIKV infection in TM cells. We show that ZIKV activates the LKB1-AMPK-ACC signaling axis, positioning AMPKα as a central node in the host response to infection. AMPKα is a master regulator of cellular energy homeostasis that integrates metabolic stress with immune signaling. Prior studies have shown that ZIKV suppresses AMPKα activity in retinal vascular endothelial cells [18], whereas other viruses, including DENV and hepatitis C virus, modulate AMPK signaling to support their replication [46,47]. Here, we demonstrate that ZIKV induces robust, time-dependent activation of AMPKα in TM cells, highlighting cell-type-specific differences in metabolic responses within the eye. Functionally, AMPK activation limits ZIKV replication. Pharmacological activation using AICAR, metformin, or 2DG significantly reduced ZIKV replication in TM cells, whereas inhibition of AMPK with dorsomorphin enhanced viral propagation. Mechanistically, AMPKα activation promoted innate antiviral responses, including induction of PRRs (TLR-3, RIG-I, MDA5), type I and II IFNs (IFN-α1, IFN-β2, IFN-γ), and ISGs (ISG-15, OAS2, MX1). At the same time, AMPK activation attenuated the expression of pro-inflammatory cytokines and chemokines, such as IL-6, IL-1β, and CCL3, suggesting a dual role in promoting antiviral defense while limiting immunopathology. These findings are consistent with previous studies linking AMPK to interferon signaling, NF-κB regulation, and STING-dependent antiviral responses [15,16,17], highlighting its broader role at the intersection of metabolism and immunity.

A notable and unexpected finding of this study is that ZIKV-induced AMPKα activation does not suppress lipid biosynthesis, contrary to predictions from canonical AMPK signaling. Instead, ZIKV drives a coordinated upregulation of lipogenic and lipid-storage pathways, including activation of SREBP1, FASN, DGAT1, and perilipin-3, leading to robust LD accumulation. Concurrently, fatty acid β-oxidation is enhanced, as indicated by increased CPT1A expression. DGAT1 catalyzes the final step in triacylglycerol synthesis, whereas CPT1A regulates mitochondrial fatty acid oxidation for ATP production. We observed simultaneous upregulation of both DGAT1 and CPT1A by ZIKV in TM cells. This apparent uncoupling of AMPK activation from its canonical anti-lipogenic effects suggests that ZIKV reprograms host metabolism to maintain a highly dynamic lipid environment. Such metabolic flexibility likely supports both the biosynthetic demands of viral replication and the energetic requirements of infected cells. Our findings corroborated other studies showing similar metabolic reprogramming in neural stem cells, neuroblastoma cells, and retinal pigment epithelial cells during ZIKV infection, as well as in other flaviviruses, such as DENV and hepatitis C virus, which exploit lipid droplets for replication complex formation [35,36,37,39].

Functionally, our data demonstrates that both LD biogenesis and β-oxidation are essential for ZIKV replication in TM cells. Pharmacological inhibition of DGAT1 or CPT1A significantly reduced LD formation and viral replication, confirming that ZIKV depends on both lipid storage and energy-generating pathways. Our findings are consistent with studies showing that DGAT1 inhibition limits replication of ZIKV in neural stem cells and associated inflammatory response in mice [36]. Similarly, DGAT1 inhibition has been shown to limit DENV, hepatitis C virus, and SARS-CoV-2 [37,39,40,41], while CPT1A inhibition has been shown to inhibit DENV replication [42]. Notably, inhibition of these pathways also reduced downstream antiviral and inflammatory responses in TM, likely reflecting decreased viral burden. Together, these results highlight that LD serve as multifunctional platforms during ZIKV infection, supporting viral replication while shaping host immune responses.

In contrast to the proviral role of endogenous lipid metabolism, exogenous fatty acid supplementation revealed a strong dependence on lipid composition. Polyunsaturated fatty acids, such as oleic and linoleic acids, markedly inhibited ZIKV replication, whereas the saturated fatty acid palmitate promoted viral replication. Mechanistic studies showed that unsaturated fatty acids impair early stages of the viral life cycles, including viral attachment and entry, and also exhibit direct virucidal activity, likely through disruption of viral envelope integrity. Our findings are consistent with prior reports demonstrating that unsaturated fatty acids can destabilize viral lipid membranes and interfere with viral fusion, including those of ZIKV, DENV, HSV, IAV, and SARS-CoV-2 [48,49]. Similarly, unsaturated fatty acids, such as oleic acid and linoleic acid, have been shown to inactivate enveloped viruses such as HSV, IAV, and Sendai viruses [50]. Importantly, these antiviral effects occurred despite increased LD accumulation, indicating that lipid composition, rather than total lipid abundance, is a key determinant of infection outcome. The differential effects of saturated and unsaturated fatty acids further highlight the complexity of host–virus metabolic interactions. While saturated fatty acids can promote lipid droplet formation and potentially support viral replication, unsaturated fatty acids appear to create a hostile membrane environment that limits viral infectivity. This distinction helps reconcile conflicting observations on the role of fatty acids in viral infections and underscores the importance of lipid composition in shaping viral life cycles. Targeted modulation with specific fatty acids, as well as inhibition of fatty acid synthesis, has been shown to confer physiological benefits by limiting viral replication. For instance, linoleic acid has been reported to inhibit cellular entry and reduce replication of SARS-CoV-2 [51]. Similarly, ω-3 fatty acids such as DHA and EPA can modulate host immune responses, attenuating virus-induced cytokine production, and may disrupt the formation of viral replication complexes by altering membrane lipid composition [51,52]. In addition, inhibition of FASN with drugs such as orlistat or TVB-3166 reduces replication of viruses, including SARS-CoV-2, RSV, and HCV, by limiting the availability of palmitate required for viral replication [53,54].

Importantly, the conclusion drawn from our in vitro experiments using primary HTMC, together with in vivo studies, underscore the translational relevance of these findings. In vivo, modulation of metabolic pathways through AMPK activation, inhibition of lipid droplet formation (DGAT1) or β-oxidation (CPT1A), or supplementation with unsaturated fatty acid (linoleic acid) significantly reduced ZIKV-induced ocular pathology and inflammation. These interventions not only limited viral replication but also preserved retinal structure, highlighting their therapeutic potential. Notably, AMPK activation provided dual benefits by enhancing antiviral immunity while suppressing excessive inflammation, a critical consideration in preventing tissue damage in the eye. Consistent with our findings, previous studies have shown that AMPK activation promotes antiviral responses, while inhibition of LD biogenesis or β-oxidation pathways reduces virus-induced inflammation [21,23,36,55].

Collectively, our findings support a model in which ZIKV induces a metabolically reprogrammed state characterized by concurrent activation of AMPK and lipid metabolic pathways. While AMPK activation represents a host defense mechanism that restricts viral replication and enhances immune responses, ZIKV counteracts this effect by driving lipid synthesis, storage, and oxidation to sustain its replication. The balance between these opposing processes ultimately determines viral fitness and disease outcome. These insights have broader implications for antiviral strategies, as targeting host metabolic pathways may offer a robust approach that is less prone to resistance than direct-acting antivirals. Although we used pharmacological modulators to investigate the roles of DGAT1 (LD biogenesis) and CPT1A (β-oxidation), future studies using loss-of-function approaches are required to validate these findings and to exclude any potential off-target effects of chemical inhibitors. In addition, further work is needed to elucidate the molecular mechanisms by which ZIKV uncouples AMPK signaling from its canonical anti-lipogenic functions and to explore lipid-targeted therapeutic strategies to limit viral infection.

## 5. Conclusions

Our study demonstrates that ZIKV reprograms host energy and lipid metabolism in TM cells to promote viral replication. ZIKV-induced LD biogenesis creates a favorable environment for viral replication, whereas activation of AMPK or inhibition of DGAT1 and CPT1A exerts protective effects by limiting viral replication. Together, our study establishes host metabolic pathways as key determinants of ZIKV pathogenesis in the TM and identifies potential metabolic targets for therapeutic intervention to mitigate ZIKV-induced ocular complications.

## Data Availability

The original contributions presented in this study are included within the manuscript and associated figure files. Further inquiries can be directed to the corresponding author.
